# From recommendation to action: psychosocial factors influencing physician intention to use Health Technology Assessment (HTA) recommendations

**DOI:** 10.1186/1748-5908-1-8

**Published:** 2006-03-31

**Authors:** Marie-Pierre Gagnon, Emília Sánchez, Joan MV Pons

**Affiliations:** 1 Evaluative Research Unit, Quebec University Hospital Centre, Quebec, Canada; 2 Catalan Agency for Health Technology Assessment and Research (CAHTAR), Barcelona, Spain

## Abstract

**Background:**

Evaluating the impact of recommendations based upon health technology assessment (HTA) represents a challenge for both HTA agencies and healthcare policy-makers. Using a psychosocial theoretical framework, this study aimed at exploring the factors affecting physician intention to adopt HTA recommendations. The selected recommendations were prioritisation systems for patients on waiting lists for two surgical procedures: hip and knee replacement and cataract surgery.

**Methods:**

Determinants of physician intention to use HTA recommendations for patient prioritisation were assessed by a questionnaire based upon the Theory of Interpersonal Behaviour. A total of 96 physicians from two medical specialties (ophthalmology and orthopaedic surgery) responded to the questionnaire (response rate 44.2%). A multiple analysis of variance (MANOVA) was performed to assess differences between medical specialties on the set of theoretical variables. Given the main effect difference between specialties, two regression models were tested separately to assess the psychosocial determinants of physician intention to use HTA recommendations for the prioritisation of patients on waiting lists for surgical procedures.

**Results:**

Factors influencing physician intention to use HTA recommendations differ between groups of specialists. Intention to use the prioritisation system for patients on waiting lists for cataract surgery among ophthalmologists was related to attitude towards the behaviour, social norms, as well as personal normative beliefs. Intention to use HTA recommendations for patient prioritisation for hip and knee replacement among orthopaedic surgeons was explained by: perception of conditions that facilitated the realisation of the behaviour, personal normative beliefs, and habit of using HTA recommendations in clinical work.

**Conclusion:**

This study offers a model to assess factors influencing the intention to adopt recommendations from health technology assessment into professional practice. Results identify determinant factors that should be considered in the elaboration of strategies to support the implementation of evidence-based practice, with respect to emerging health technologies and modalities of practice. However, it is important to emphasise that behavioural determinants of evidence-based practice vary according to the specific technology considered. Evidence-based implementation of HTA recommendations, as well as other evidence-based practices, should build on a theoretical understanding of the complex forces that shape the practice of healthcare professionals.

## Background

Health Technology Assessment (HTA) is a multidisciplinary field of applied research that aims to provide the best evidence available on health technologies in order to inform policy-making [1,2]. In HTA, the definition of *health technology *is broad and encompasses all methods used by health professionals to promote health, prevent and treat disease, and improve rehabilitation and long-term care [3].

It is generally recognised that there is a gap between the production of scientific evidence and its utilisation to inform decision-making, [4], and this also applies to the field of HTA [5-8]. Despite growing interest in HTA, both in the governmental and scientific spheres, few efforts have been made to assess HTA impact on decision-making at different levels of the healthcare system [6]. Furthermore, there is a paucity of specific methodologies and tools to assess the uptake of HTA recommendations [5].

At the health policy level, previous work has reported that HTA recommendations could influence decision-making [9-11]. According to a multi-method study of the implementation of guidance issued by the National Institute for Clinical Excellence (NICE) in England and Wales, [12] the extent to which HTA led to changes in practices was variable. Moreover, a review of HTA utilisation in four European countries indicates that, in spite of substantial human and financial investments, the actual impact of HTA on policy-making was still limited [13].

Hivon and collaborators have explored end-users' perceptions and use of HTA recommendations [14]. Their findings indicate that knowledge produced from HTA was not always used directly in decision-making, but could serve various purposes. According to these authors, HTA recommendations could have an *instrumental*, *conceptual *or *symbolic *use in decision-making [14]. Instrumental use implies that recommendations from HTA are directly translated into a decision. HTA recommendations also can have a conceptual use by providing a knowledge basis for debate and positioning. Finally, decision-makers can make a symbolic use of HTA recommendations, using them to reinforce or justify their decisions. Thus, studies assessing HTA utilisation should explore the various purposes that scientific evidence can serve in the formulation of healthcare policies.

At the healthcare organisations level, the implementation of hospital-based HTA activities could represent a strategy to improve practices [7]. Hospital-based HTA is believed to provide scientific evidence that is context-relevant, which would eventually lead to the adoption of best practices [15,16]. Experiences with HTA activities in hospitals have reported positive impact on resources and costs [15]. Other experiences of decentralized HTA activities include the implementation of units dedicated to HTA at the regional health authority level, such as in Health Regions in Canada [17]. However, evidence is still lacking on how HTA activities should be integrated within healthcare organisations [18].

Until now, little is known about the implementation of HTA recommendations at the individual level, i.e. in the daily practice of healthcare professionals. However, the literature on physician adoption of scientific evidence and interventions to improve it is extensive [19]. Thus, it is possible to draw from this body of knowledge in order to better understand the mechanisms involved in the adoption of HTA recommendations into clinical practices.

### Theoretical foundations

In the field of social psychology, various theories and models have been proposed to understand what influences the adoption of behaviours. Triandis' Theory of Interpersonal Behaviour (TIB) [20] encompasses many of the behavioural determinants found in other psychosocial theories, such as the Theory of Planed Behaviour [21] and the Social Cognitive Theory [22]. Moreover, the TIB also considers cultural, social, and moral factors that are particularly important in the study of specific groups, such as healthcare professionals [23,24].

**Figure 1:**
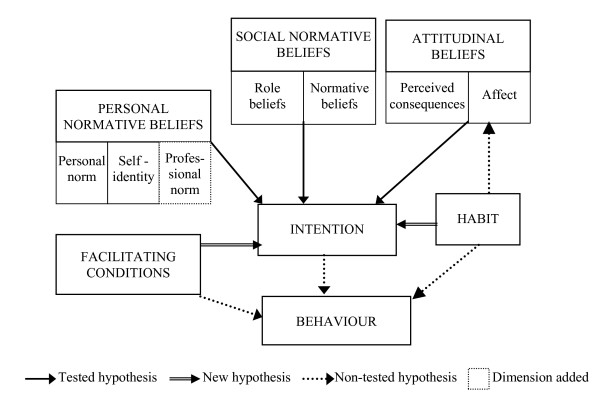
**Theoretical Model **Adapted from Triandis' Theory of Interpersonal Behaviour [22]

A schema adapted from the TIB is presented in Figure 1. According to this theory, human behaviour is formed by three components: intention, facilitating conditions, and habit. *Intention *refers to the individual's motivation regarding the performance of a given behaviour. *Facilitating conditions *represent perceived factors in the environment that can ease or impede the realisation of a given behaviour. *Habit *refers to how routine a given behaviour has become, i.e. the frequency of its occurrence. Habit directly influences the behaviour, but can also have an influence on affect. However, this hypothesis was not tested in the present study.

In the TIB, the behavioural intention is formed by attitudinal as well as normative beliefs. Attitudinal beliefs comprise two dimensions: affect and perceived consequences. *Affect *represents an emotional state that the performance of a given behaviour evokes for an individual. It is considered as the affective perceived consequences of the behaviour, whereas *perceived consequences *refer to individual's perception of the instrumental consequences of the behaviour.

The TIB also distinguishes between two normative dimensions: social and personal. Social normative beliefs are formed by normative and role beliefs. *Normative beliefs *consist of the internalisation by an individual of referent people's or groups' opinions about the realisation of the behaviour, whereas *role beliefs *reflect the extent to which an individual thinks someone of his or her age, gender, and social position should or should not behave. With respect to the personal normative beliefs, *personal norm *represents the feeling of personal obligation regarding the performance of a given behaviour, whereas *self-identity *refers to the degree of congruence between the individual's perception of self and the characteristics associated with the realisation of the behaviour.

For the purpose of this study, modifications were brought to the original TIB model. These modifications were consistent with a previous study that has adapted the TIB to understand healthcare professional behaviour [24]. First, the dependent variable of interest in this study is the behavioural intention rather than the behaviour. Thus, the original relationships between facilitating conditions and behaviour, as well as between habit and behaviour have been modified to explore the influence of these constructs on the behavioural intention. These relationships are consistent with previous studies that used the TIB to predict behavioural intention [25-27].

Furthermore, in an effort to better adapt the TIB to health professional behaviour, another dimension was added to the personal normative beliefs – the *professional norm*. This variable is related to the integration by the self of the specific normative pressures of one's professional group. The medical profession has a particular culture and sets of norms (e.g. the Hippocratic Oath) that also influence individual physician behaviour [28]. In a previous study, adding the professional norm to the personal normative construct significantly improved the predictive validity of this construct in explaining physicians' decision to adopt a new technology [24]. This construct is also consistent with the concept of *collective self*, as proposed by Triandis, which corresponds to the individual's assessment of how she or he should behave given her or his belonging to a specific reference group [29]. The professional norm is considered a of the dimension of the personal normative construct since previous work has shown association between these factors [24].

To the best of our knowledge, the TIB has not previously been applied to the study of the adoption of evidence-based recommendations into medical practice. However, this model was successful in explaining a variety of professional behaviours, such as the adoption of information and communication technologies [24,25,30,31].

### Description of the study

This study is part of a larger initiative aimed at applying a multi-dimensional theoretical framework to assess the impact of HTA recommendations on decision-making at different levels of the healthcare system. Thus, various methods were used in order to assess factors influencing the uptake of HTA recommendations at the healthcare organisation and clinical decision-making levels. HTA adoption at the organisational level was assessed through a qualitative approach by means of interviews and observations at 15 hospitals of Catalonia. The results of the qualitative study are presented elsewhere [32,33].

In summary, the qualitative study indicates that factors related to the organisation and financing of the health system influence adoption of HTA recommendations at the hospital level. Furthermore, collaborations between hospitals and the HTA agency favour the integration of recommendations into organisational practices. At the professional level, the high degree of autonomy of medical specialists, the importance of peers and collegial control, and the definition of professional roles and responsibilities influence adoption of HTA recommendations.

The present article focuses on the impact of HTA recommendations at the individual level, which has been conceptualised as physician intention to use HTA recommendations to support clinical decision-making. This study was conducted as part of a postdoctoral fellowship (MPG), and the research protocol was approved by Catalonia and Quebec governments. The Catalan Agency for Health Technology Assessment and Research (CAHTAR) also reviewed the research protocol and provided support for the study.

### Research Questions

Based upon the TIB, this study aimed to answer the following questions:

1. Which psychosocial factors from the TIB (attitudinal beliefs, social normative beliefs, personal normative beliefs, facilitating conditions, habit) significantly explain the intention of physicians to adopt these recommendations into their practice?

2. Are the psychosocial factors influencing physicians' intention to adopt HTA recommendations significantly different between the two groups of specialists?

3. Do sociodemographic and professional factors (age, gender, work experience) influence physicians' intention to adopt HTA recommendations over and above the psychosocial constructs from the TIB?

## Methods

### Selection of health technologies

A consensus was reached with researchers from the CAHTAR to select which recommendations would be investigated. The criteria used in the selection were: 1) publication time sufficient for the HTA recommendation to have been largely disseminated; 2) recommendations representing administrative and clinical health technologies, since the literature reports important variations in factors affecting the adoption of these two types of innovations;[34] and 3) similar recommendations that would allow comparisons between cases for a greater internal validity. Thus, a total of three recommendations were selected. Two were related to clinical-administrative technologies, namely prioritisation systems for patients on waiting lists for two distinct surgical procedures – cataract surgery and hip and knee replacement. The third recommendation covered the prescription of external pump for continuous subcutaneous insulin infusion for patients with Type I diabetes. However, it was not possible to analyse the factors affecting the adoption of this recommendation quantitatively, given the limited number of endocrinologists (7) in the sample. Thus, only the recommendations regarding the two prioritisation systems were considered in the analysis of HTA recommendations' impact at the individual decision-making level.

Both recommendations proposed a scoring system to assess patient priority on waiting lists for the targeted surgical procedures. The prioritisation systems for cataract surgery and hip and knee replacement were similar, although specific scoring items were used. Their utilisation by physicians practicing in the Catalan network of public hospitals was made mandatory through an instruction issued by the Servei Català de la Salut (the Catalan Health Service) in November 2004.

### Development of the survey instrument

The field of social psychology has a long tradition in the development of survey instruments based upon theoretical frameworks. In the present study, questionnaire development was based on several theorists' recommendations [21,35,36]. The TIB provided the conceptual constructs that were measured, but we adapted the content (i.e. wording of the questions) to the specific behaviour under study and the particular culture of the target group. This is known in anthropology as the *emic-etic *approach and has been recommended by psychosocial theorists in order to ensure the cultural sensitivity of a study [37,38].

First, an open-ended questionnaire was prepared in order to assess the modally salient beliefs in the study population with respect to the behaviour under consideration. Salient beliefs are the first responses to come to a respondent's mind when asking an open-ended question. Therefore, modally salient beliefs are the most frequently reported beliefs regarding the attributes of performing a particular behaviour in the target group [39]. Thus, a purposive sample of 10 physicians within each medical specialty was sent a questionnaire comprising eight open-ended questions. Questions assessed the attitudinal, social normative and personal normative beliefs, as well as the perceived facilitating conditions and barriers with respect to using HTA recommendations to support decision-making.

Completed questionnaires were received from five ophthalmologists and seven orthopaedic surgeons. Responses were compiled for each specialty. A content analysis was performed to classify responses into thematic categories. Then the number of responses in each category was compiled, and those having a frequency of two or more were kept as the modally salient beliefs. These salient beliefs were used as the items to assess each theoretical construct of the TIB. A specific questionnaire was developed for each medical specialty, since two distinct recommendations were addressed. However, given the similitude between these recommendations, the two questionnaires used the same items to assess theoretical constructs, thus allowing for the combination of results and comparisons between groups.

The first page of the questionnaire presented the study and gave instructions to participants. A sentence indicated that returning the questionnaire implied informed consented to participate in the study. The questionnaire began with a vignette describing a clinical case for which the surgical procedure (cataract surgery or hip and knee replacement) was relevant. By referring to the case presented in the vignette, physicians were asked to answer a total of 30 questions measuring the theoretical constructs of the TIB.

Each theoretical item was assessed by a question measured on a five-point Likert scale. For example, to what extent do you agree with the following affirmation – "*It would be easy for me to use CAHTAR's recommendations to support my decision in this case.*" 1) Totally disagree; 2) Slightly disagree; 3) Neither agree nor disagree; 4) Slightly agree; or 5) Totally agree. The only exception was for the items composing the attitudinal construct that were assessed by means of 5-point bi-polar adjective scales. For example, "*For me, using CAHTAR's recommendations to support decision-making in this case would be...*" 1) Very foolish; 2) Somewhat foolish; 3) Neither foolish nor wise; 4) Somewhat wise; or 5) Very wise. The number of items used to assess each theoretical construct and their internal consistency are provided in Table 1. The Cronbach α was used to verify the internal consistency of theoretical constructs. As shown in Table 1, all constructs showed satisfactory internal consistency, with Cronbach α higher than 0.70 [40].

**Table 1:** Internal consistency of theoretical constructs

Construct	Number of items	Internal consistency (Cronbach's alpha)
Intention	3	0.85
Attitudinal beliefs	7	0.81
Personal normative beliefs	6	0.86
Social normative beliefs	6	0.82
Facilitating conditions	3	0.75
Habit	4	0.87

Finally, socio-demographic information (*age group*, *gender*, *years of clinical experience*, and *medical specialty*) was collected at the end of the questionnaire. The questionnaire was pre-tested with two physicians of each specialty in order to assess face validity and duration. Subsequently, minor adjustments were done to the wording of some questions. The questionnaire took approximately 15 minutes to complete.

### Participants and setting

A total of 15 hospitals were selected to most fully represent the various profiles of Catalan hospitals. Hospitals from the eight Catalan Health Regions were represented. The sample consisted of publicly and privately-funded hospitals (all provided services in the public system), as well as large teaching hospitals and smaller general hospitals. Heads of department or service for the targeted specialties (ophthalmology and orthopaedic surgery) were identified in each hospital as the local collaborators. The principal investigator contacted them by telephone to describe the study and solicit their participation. After receiving consent from all contacted persons, a package containing study questionnaires corresponding to the number of physicians who worked in the service was delivered to the local collaborator in each hospital. The total sample consisted of 217 physicians (80 ophthalmologists and 137 orthopaedic surgeons).

### Statistical analyses

First, descriptive analyses of distribution were conducted. Correlations between theoretical variables and between theoretical and external variables were assessed and are reported in Table 2. All the theoretical constructs from the TIB had a significant positive association with the intention. Medical specialty was the only external variable having a significant correlation with theoretical variables. None of the external variables were significantly correlated with intention.

**Table 2:** Zero-order correlations between theoretical and sociodemographic variables

Variable	Attitude	Personal norms	Social norms	Facilitating cond.	Habit	Age	Gender	Specialty	Experience
Intention	0.715***	0.781***	0.716***	0.510***	0.677***	0.109	-0.003	-0.049	0.147
Attitude		0.664***	0.754***	0.500***	0.591***	-0.046	0.035	-0.303**	-0.038
Personal norms			0.721***	0.424***	0.695***	0.044	0.093	-0.241*	0.063
Social norms				0.434***	0.666***	-0.033	0.085	-0.253*	-0.007
Facilitating cond.					0.422***	-0.098	0.022	-0.136	-0.092
Habit						0.106	0.020	-0.196	0.135
Age							-0.283*	0.422***	0.903***
Gender								-0.436***	-0.278**
Specialty									.0403***

* p < 0.05; ** p < 0.01; *** p < 0.001

Second, a comparison between the two groups of specialists was performed on the set of theoretical variables using the multivariate analysis of variance (MANOVA). Given the significant differences between groups, two independent hierarchical regression models were tested in order to assess the determinants of physician intention to use HTA recommendations. The potential impact of external variables (socio-demographic and professional characteristics) on intention was tested following Pedhazur's recommendation, which consists of comparing the R^2 ^of the model containing only theoretical variables with the R^2 ^of a model also containing external variables [41]. No significant difference was found. We also assessed potential interaction effects of external variables by entering interaction terms between theoretical and external variables that were significantly correlated (e.g. attitude and experience in the orthopaedic surgeons group) in the regression equation [42], but no significant effect was found for the interaction terms. The final regression models were calculated by keeping only the significant predictors in the equation. All statistical analyses were performed using SPSS version 12.0. (SPSS Inc., Chicago, IL)

## Results

### Descriptive statistics

A total of 96 physicians returned completed questionnaires (35 ophthalmologists and 61 orthopaedic surgeons) for a global response rate of 44.2%. Table 3 presents the sociodemographic and professional characteristics of participants. There are significant differences between the two groups of specialists. First, gender distribution is uneven, since women are generally a minority in orthopaedic surgery. Second, age distribution also is different between the two specialties, orthopaedics surgeons being older than ophthalmologists. Likewise, the mean clinical experience is higher among orthopaedic surgeons. These differences probably reflect a trend for specialty choice in younger cohorts of physicians where the proportion of women is higher [43].

**Table 3:** Sociodemographic and professional characteristics of respondents

Variable	Medical specialty		Difference (*chi-square *or *Student t-test*)
	Ophthalmology	Orthopaedic surgery	
**Gender**			
Male (%)	19 (54.3)	51 (83.6)	χ^2 ^= 15.20 p < 0.001
Female (%)	9 (25.7)	1 (1.6)	
Missing (%)	7 (20.0)	9 (14.8)	
**Age group**			
< 30 years (%)	10 (28.6)	3 (4.9)	χ^2 ^= 19.95 p < 0.001
30 – 39 years (%)	12 (34.3)	9 (14.8)	
40 – 49 years (%)	8 (22.9)	24 (39.3)	
≥ 50 years (%)	5 (14.3)	25 (41.0)	
**Clinical experience**^a^			
Mean	11.0	18.7	*t *= 4.22
Standard deviation	± 8.4	± 8.8	p < 0.001

^a ^Two missing values

Table 4 reports the descriptive statistics (means and standard deviations) of the theoretical variables. Normality of distribution and possible collinearity were assessed and results were satisfactory (see the research report for detailed results [32]). The mean value of the intention to use HTA recommendations is not markedly different between groups. However, all theoretical variables have a higher mean among ophthalmologists. The majority of theoretical variables have a mean value higher than 3, which corresponds to a positive value. One exception is the variable habit that has a negative value (lower than 3) in both groups. Moreover, personal and social normative beliefs have a negative value among orthopaedic surgeons. These findings indicate that there might be significant differences between the two groups of specialists.

**Table 4:** Main effect difference and differences in theoretical variables between medical specialties

Theoretical variable	Ophthalmology (n = 35) Mean (sd)	Orthopaedic surgery (n = 61) Mean (sd)	F-test for univariate difference (*df*)	*p *value*
Intention	3.59 (± 1.13)	3.49 (± .87)	F = 0.23 (*1*, *94*)	0.63
Attitudinal beliefs	3.68 (± .65)	3.23 (± .72)	F = 9.50 (*1*, *94*)	0.003
Personal normative beliefs	3.38 (± 1.14)	2.91 (± .77)	F = 5.80 (*1*, *94*)	0.018
Social normative beliefs	3.38 (± .80)	2.97 (± .74)	F = 6.43 (*1*, *94*)	0.013
Facilitating conditions	4.26 (± .75)	3.59 (± 1.15)	F = 9.45 (*1*, *94*)	0.003
Habit	2.75 (± 1.24)	2.31 (± .96)	F = 3.75 (*1*, *94*)	0.056

Hotelling's Trace = 0.336 [F (6, 89) = 4.98; p < 0.0001]				

* Considered significant at p < 0.05

### Differences in intention to use HTA recommendations between specialties

To assess the main effect difference between the two groups, i.e. how they globally differ on the set of theoretical variables, a multivariate analysis of variance (MANOVA) was conducted. This test allows for verifying equality of variances between multiple variables at the same time, without having to adjust for multiple testing. According to Hair et al.,[44] a MANOVA can be performed for uneven groups if the following three conditions are met: 1) the number of observations in the smallest group is higher than the number of dependent variables; 2) the number of observations in each group is higher than 20; and 3) there is a minimum of five observations for each dependent variable. All three conditions were met in this case.

The Hotelling's Trace was used to assess the main effect of medical specialty on the set of theoretical variables, as it has been recommended for two-groups MANOVA. [45] As shown in Table 4, the Hotelling's Trace test is significant, indicating that there is a global difference between groups. Furthermore, univariate tests show that all explicative variables of the model also are significantly different, except habit. However, the dependent variable of the model (intention) is not significantly different between groups.

Therefore, given that intention to use HTA recommendations to support decision-making might have had different determinants within each group of medical specialists, two logistical regression models were tested, including variables from the TIB and external variables.

### Factors influencing intention to use HTA recommendations for cataract surgery

Table 5 presents the final regression model of the intention to use HTA recommendations for prioritisation of patients on waiting lists for cataract surgery. The model was significant and explained 87% of the variance (adjusted R^2^) in ophthalmologists' intention to use the HTA recommendations to support decision-making. The three determinants explaining this intention were, in order of importance: attitudinal beliefs (β = 0.40), personal normative beliefs (β = 0.36), and social normative beliefs (β = 0.25).

**Table 5:** Regression of the intention to use HTA recommendations for prioritisation of patients on waiting lists for cataract surgery

Theoretical variable	Standard estimate (β)	*p *value*
Attitudinal beliefs	0.40	0.001
Personal normative beliefs	0.36	0.004
Social normative beliefs	0.25	0.044

R^2 ^of the model: 0.89 [F (3, 31) = 77.44; p < 0.001] ; Adjusted R^2 ^= 0.87		

* Considered significant at p < 0.05

### Factors influencing intention to use HTA recommendations for hip and knee replacement

The final regression model tested to explain the intention to use HTA recommendations for prioritisation of patients on waiting lists for hip and knee replacement is reported in Table 6. Again, the regression model was significant and explained 65% of the variance (adjusted R^2^) in orthopaedic surgeons' intention to use the recommendations to support decision-making. The strongest predictors were facilitating conditions (β = 0.39), personal normative beliefs (β = 0.38), and habit (β = 0.25).

**Table 6:** Regression of the intention to use HTA recommendations for prioritisation of patients on waiting lists for hip and knee replacement

Theoretical variable	Standard estimate (β)	*p *value*
Facilitating conditions	0.39	0.000
Personal normative beliefs	0.38	0.000
Habit	0.25	0.039

R^2 ^of the model: 0.66 [F (3, 57) = 37.40; p < 0.001] ; Adjusted R^2 ^= 0.65		

* Considered significant at p < 0.05

## Discussion

This study was the first, to the best of our knowledge, to assess the psychological factors influencing physician intention to use HTA recommendations based upon a recognised theoretical framework. The TIB has been successful in explaining a variety of human behaviours, including the adoption of health technologies among healthcare professionals [24,25,30,31]. Using an established theoretical framework to assess the determinants of professional behaviours presents at least four advantages. First, it provides a basis for comparison between similar studies, thus supporting knowledge development in the field [46]. Second, it offers a sound methodological approach that improves the internal validity of studies based upon the advances in social psychology measurement. Third, it facilitates the realisation of systematic reviews in the field of implementation science [46]. Finally, it allows for the development of strategies to improve the success of interventions to implement evidence-based practices [46-49]. This study also provides support to the cultural adaptability of a psychosocial theoretical framework such as the TIB, since the items forming theoretical constructs were adapted to the specific context in which the study took place. This framework could thus be adapted and applied to a variety of settings in the field of implementation science.

A major finding of this study is that intention of physicians to use HTA recommendations in their practice is influenced by a different set of psychosocial factors, depending on the specific context. This difference can either be attributed to the characteristics of the technology targeted in the HTA recommendations, the social and cultural characteristics of the medical specialty, the specific context in which recommendations are implemented, or a combination of these factors. It would be necessary to study the adoption of various HTA recommendations across different medical specialties and contexts in order to verify these hypotheses.

Nevertheless, the present study supports the need for mapping interventions to specific population groups in order to improve the adoption of evidence-based practices [50]. A previous study has reported limited impact of a tailored intervention aimed at introducing evidence-based practices among physicians,[51] but this lack of success was largely due to problems related to the implementation of the intervention [52].

Among the factors that were associated with intention to use HTA recommendations to support decision-making, personal normative beliefs were important in both groups of specialists. This variable was formed by three components, namely, personal norm, self-identity, and professional norm. The impact of personal morals or principles on clinical behaviours has been reported in a cross-cultural study of physicians' intention to prescribe hormone therapy [46]. The construct of professional norm, added to the TIB framework for this study, was found to influence physician intention to adopt telemedicine [24]. However, this is a relatively new concept that needs further psychometrical developments.

The influence of attitudinal beliefs on the intention to use HTA recommendations was significant only in the ophthalmologists group. Attitude has been found as an important determinant of clinical behaviours in other studies [12,53]. However, attitude was not associated with the intention of physicians to adopt telemedicine [24]. Thus, a positive perception of the benefits of using HTA recommendations was more important in explaining the intention to adopt the prioritisation system for cataract surgery. Borrowing a concept from the diffusion of innovation theory,[54] ophthalmologists who had the intention to use the prioritisation system were those who perceived a relative advantage to this innovation [55]. Hence, the decision to use the prioritisation system or not was mostly perceived as an individual choice. One plausible explanation is the fact that waiting lists were not perceived as a big issue for ophthalmologists, since most of them also performed cataract surgery in private practice. Thus, external pressure to adopt the prioritisation system was not as strong as for hip and knee replacement.

Facilitating conditions, i.e. factors in the environment that support the realisation of the behaviour, were the most influential determinant of intention to use HTA recommendations among orthopaedic surgeons. One plausible explanation is the fact that hip and knee replacements are more complex and costly procedures that require greater resources. Thus, the prioritisation system was endorsed by a majority of the departments as the 'local standard.' Furthermore, the variable *habit *was also associated with the intention to use HTA recommendations among orthopaedic surgeons, which supports the previous hypothesis. It is likely that individual healthcare professionals will tend to adopt evidence-based practice more easily when there is a supportive culture in the working environment. However, it is important to acknowledge a possible threat to professional autonomy when introducing explicit rationing policies, such as prioritisation systems for surgical procedures that can lead to resistance to change [56].

Previous studies of the impact of HTA on decision-making at the health policy level recognize the difficulty of measuring how a specific recommendation would inform decision-making on a given topic [57,58]. Another contribution of this study is that it proposes a strategy to assess the impact of HTA recommendations at the clinical decision-making level. Of course, using behavioural intention as the dependent variable is a proxy for estimating actual behaviour, but the literature generally supports the concordance between intention and subsequent behaviour [59]. Recent efforts have been made to bridge the 'intention-behaviour gap.' For instance, moral factors, such as anticipated regret and moral norm have a significant impact on the consistence between intention and subsequent behaviour [60,61]. However, longitudinal studies are needed to assess the correspondence between physicians' intention to adopt evidence-based practices and their subsequent behaviours.

### Limitations of the study results

Among the factors that may affect the possibility to generalise the results, it is important to mention a possible participation bias since respondents may have been more knowledgeable and/or interested in the HTA recommendations under study than non-respondents. Unfortunately, contacting non-respondents to assess this potential bias was not feasible since the study was anonymous.

The sample size was limited, despite a satisfactory response rate for this specific population. Previous studies usually report lower response rates for mail surveys among physicians [62,63]. The involvement of the Head of Department from each specialty in participating hospitals appeared as a successful strategy to improve participation in the study.

Given the small sample size, especially in the ophthalmologists group, it is important to use caution when interpreting the results. For undersized samples, the risk of unstable solution is greater when the independent variables are highly correlated. Also, a high R^2 ^may reflect a problem of 'over-fitting,' i.e. a perfect but meaningless solution [64]. To test the stability of the solution in the ophthalmologists group, we verified if the pattern of the regression equation was affected by deleting the weakest predictor (social normative beliefs). The regression equation with the remaining two predictors (attitude and personal normative beliefs) was similar, indicating that the solution was stable. A multi-collinearity diagnosis was then performed. The variance inflation factors associated with independent variables were all below 10, showing no multicollinearity problem [44].

With respect to the possibility of over-fitting, other studies, both with small or larger samples, have reported high R^2 ^in the prediction of behavioural intention among healthcare professionals based upon psychosocial theories [24,46,65]. In a study of physician intention to adopt telemedicine (n = 506), a high R^2 ^(.81) also was found, and similar correlations between the independent variables were present [24]. Thus we can conclude that the solution is likely to reflect a true relationship between the psychosocial predictors from the TIB and physician intention to use HTA recommendations.

A short vignette was used to bring physicians into a decision-making situation for which their intention to refer to the HTA recommendation was assessed. The vignette contained limited information on the clinical case and a hypothetical bias might have been present [66]. However, clinical vignettes are considered a valid and comprehensive method to asses the process of care provided in actual clinical practice [67]. Therefore, the findings of this study are likely to apply to 'real life' decision-making situations.

## Conclusion

This study demonstrates the application of a social psychological model to understand the determinants of the adoption of evidence-based practices in healthcare. Of course, this represents only a small portion of the efforts needed to implement evidence-based interventions in order to improve quality in healthcare. Further work should address the translation of knowledge gained from studies on the determinants of healthcare professional behaviours into specific intervention strategies, the successful implementation of these strategies, and the evaluation of their effects on professional behaviours and, ultimately, on the effectiveness of the healthcare system.

## Competing interests

The author(s) declare that they have no competing interests.

## Authors' contributions

ES, JMVP and MPG participated in the design of the study. ES and MPG prepared the study questionnaires. MPG contacted the participants, proceeded to data collection and performed quantitative analyses. ES and JMVP reviewed the findings and a consensus was reached between all authors for data interpretation. MPG prepared a first draft of the manuscript and all authors revised and approved the last version of the manuscript.
